# A Young Female Newly Diagnosed With Takayasu’s Arteritis Masquerading As Cerebrovascular Stroke

**DOI:** 10.7759/cureus.49292

**Published:** 2023-11-23

**Authors:** Shivam J Raval, Rosy M Laxmidhar, Divya R Patel, Fehmida Laxmidhar, Vraj Solanki

**Affiliations:** 1 Internal Medicine, Byramjee Jeejeebhoy (BJ) Medical College and Civil Hospital, Ahmedabad, IND; 2 Internal Medicine, Western Reserve Health Education/Northeast Ohio Medical University (NEOMED), Warren, USA; 3 Internal Medicine, American International Institute of Medical Sciences, Udaipur, IND

**Keywords:** cerebrovascular accident (stroke), autoimmune vasculitis, ischemic stoke, stroke in the young, takayasu disease

## Abstract

The condition known as Takayasu's disease or Takayasu's arteritis is a type of vascular inflammation that affects the large and medium arteries. It can lead to a reduction in blood flow to various parts of the body, and it can cause severe complications. Patients with this disease may not have specific symptoms, which can lead to their diagnosis not being confirmed.

Takayasu's disease is believed to be a probable cause of stroke in young patients. Although stroke is a common cause of morbidity, it is usually not an initial presentation in Takayasu's disease. In this study, a young female with left-sided hemiparesis was diagnosed with Takayasu's disease after a clinical and angiographic examination.

## Introduction

Takayasu's Arteritis or Takayasu's disease is a chronic inflammatory condition that affects the large and medium arteries, mainly the aorta. The damage to the vessel wall caused by this inflammatory condition can lead to the disappearance of the smooth muscle layer and the intimal hyperplasia, which leads to vascular stenosis in almost all patients. About 25% of patients have vascular aneurysms and dilatation. Takayasu's Arteritis usually affects people between 15 and 35 years of age. Females are more prone to develop this condition. About half of the patients have neurological involvement. In about 10%, stroke is a complication [1]. However, the occurrence of acute stroke as the initial presentation in patients with Takayasu's Arteritis is infrequent and rarely reported; therefore, there is a need to complete the evaluation of the etiology of stroke in young patients in order to initiate therapy to reduce the chance of subsequent events and to encourage appropriate follow-up for better outcomes.

Here, we present a case of a 23-year-old female with normal ESR levels who was diagnosed with Takayasu's arteritis after an attack of stroke and showed clinical improvement with immunosuppressants.

## Case presentation

A young 23-year-old Asian female with no known vascular risk factors presented with left lower limb weakness and left-sided upper arm weakness for the past week. Her history indicated progressive and gradual headaches. Headache was a new onset and she also experienced neck pain, fatigue, and abnormal vision. There was no specific pattern observed, but a blurring of vision was noted with an acute onset. She also experienced a loss of appetite for one week.

The pain radiated to the back and was accompanied by numbness and tingling in both hands and also in both legs. The patient denied using illicit drugs, tobacco products, or oral contraceptives. The patient did not have a family history of vascular or rheumatologic disorders, diabetes, hypertension, autoimmune diseases, or tuberculosis. She was not obese and her background was also unremarkable.

The patient was examined in a well-ventilated room; she appeared lethargic but was conscious and followed verbal commands well. There were no signs of icterus, edema, or pallor. She was also normotensive and afebrile, with no blood pressure difference in all four limbs. All peripheral pulses were equal and normal except the bruit over the left carotid artery.

On neurological examination, the patient was conscious and oriented but lethargic. The left upper limb (UL) and the left lower limb (LL) had hypotonia, extensor planter reflex, areflexia (deep tendon reflex {DTR}) on the left lower limb, power grade 2/5 on left upper and lower limb, and the left upper motor neuron facial palsy.

**Figure 1:**
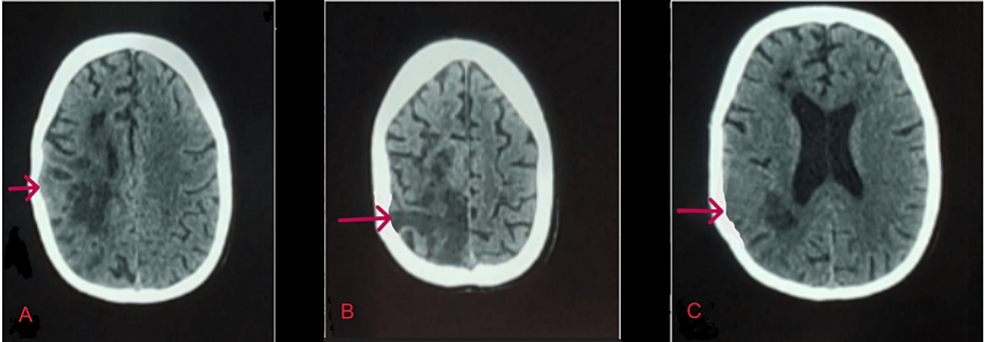
Non-contrast computed tomography of the patient. (A, B) NCCT brain showing mild hypo density with loss of gray-white matter differentiation in the right posterior frontoparietal region. (C) Chronic ischemic foci in right frontal and parietal periventricular white matter. NCCT: non-contrast computed tomography

The patient was immediately taken for non-contrast computed tomography (NCCT) brain, which showed mild hypo density with loss of gray-white matter differentiation in the right posterior frontoparietal region, suggesting acute infarct. Also showed chronic ischemic foci in the right frontal and periventricular white matter (Figures 1A-1C).

**Figure 2:**
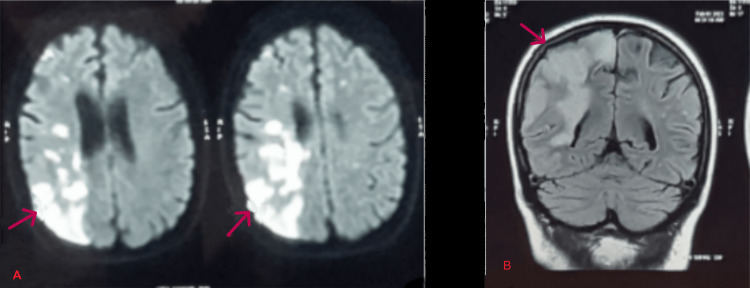
Magnetic resonance imaging of the patient. (A) Restriction on diffusion imaging involving the right posterior frontoparietal cortical-subcortical region and (B) coronal FLAIR image reveal hyperintensity in the right parietal-temporal region. ADC: apparent diffusion coefficient; DW-MRI: diffusion-weighted magnetic resonance imaging; FLAIR: fluid-attenuated inversion recovery

A magnetic resonance imaging procedure was then performed to examine the patient's brain. It revealed a multifocal area of restricted brain diffusion seen involving the right posterior frontoparietal cortical-subcortical regions (corresponding hypo intensity on apparent diffusion coefficient {ADC} maps not shown in images) (Figures 2A, 2B).

An examination of the patient's blood samples revealed that her total leukocyte count was 19,660/μL, her erythrocyte sedimentation rate was 18 mm/h, her blood urea level was 57 mg/dL, c-reactive protein (CRP) was 4.0 mg/L, lipid profile was within normal limits, homocysteine was 6.8 μmole/L. Other investigations, such as chest radiography and echocardiograms were normal. On the other hand, electrocardiography showed sinus tachycardia with a short PR interval and abnormalities in the T wave and ST waves. She was treated conservatively with aspirin and 40 mg of atorvastatin. She was stable and was able to maintain normal hemodynamics.

**Figure 3:**
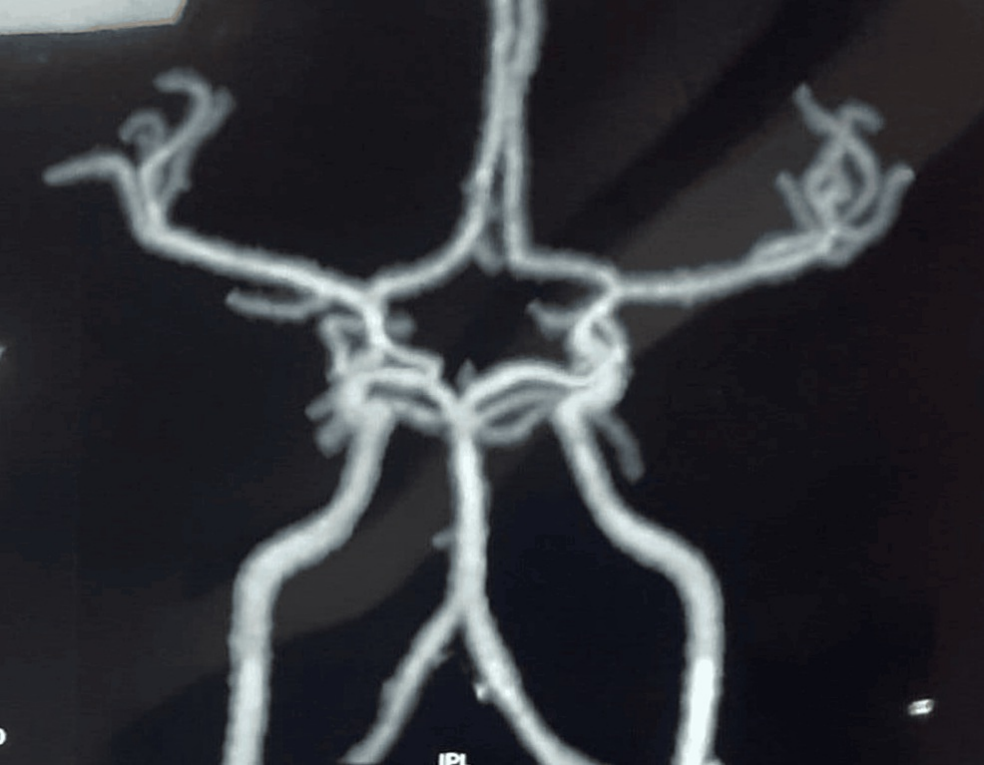
The patient's magnetic resonance angiography of extracranial arteries. The image shows poor visualization of mid-distal segments of the right common carotid artery and almost the entire left common carotid artery. MR angiography of intracranial arteries did not reveal significant stenotic vessels.

The patient's magnetic resonance angiography of extracranial arteries demonstrated poor visualization of mid-distal segment of the right common carotid artery and almost the entire left common carotid artery suggested severely diseased vessels. Magnetic resonance angiography of intracranial arteries did not reveal significant stenotic vessels (Figure 3).

Our patient met the American College of Rheumatology diagnostic criteria (three out of six). The patient was 23 years old (age of onset < 40 years) when she developed the disease. She had a history of claudication of the legs and bruit over the left carotid artery, which was not caused by fibromuscular dysplasia or arteriosclerosis. Claudication has been present for 8 to 10 days during activity, followed by weakness in the left lower and upper limbs. It was more on lifting weights and stepping on staircase. There was a narrowing of bilateral carotid arteries on MRI angiography. The diagnosis of Takayasu's arteritis was made based on the angiographic and clinical aspects.

The patient was referred to the rheumatology department for treatment. She was given oral medications, such as tablets of aspirin 75 mg/day, 40 mg/day of prednisone, 15 mg/week of oral methotrexate, and atorvastatin 40 mg per day. After three days in the hospital, she showed significant improvement. The patient's sensorium improved and she was able to talk and watch her mobile. Power was improved in her left upper limb 4/5 and lower limb 3/5 and her appetite also improved. She was then advised to continue taking the same medication for one month. After discharge, psychiatric counseling for depression due to sudden onset of paralysis was carried out, and physiotherapy was provided.

## Discussion

Younger adults are less likely to suffer from ischemic stroke than older adults. However, the underlying risk factors and pathogeneses are more diverse. About 10-15% of all strokes happen in people aged 18-50 years. Because of this, it is difficult to distinguish between stroke mimics and actual stroke cases [2]. When diagnosing patients with stroke, especially young ones, it is crucial to consider Takayasu's arteritis as a possible cause. Proper treatment is needed to minimize its progression and morbidity. In patients presenting with stroke, we usually check for signs of trauma, arrhythmia, or valvular disease. However, in our patient, none of these were present. Her echocardiography and ECG reports did not suggest any cardiac illness. The presence of a carotid artery bruit and magnetic resonance imaging provided us with a clue to her diagnosis [1].

Patients with Takayasu's arteritis may present with various symptoms, such as fever, weight loss, and sleep disorders. In the early stages, they may also experience neck or carotid pain. Later in the disease, patients may develop symptoms related to end-organ ischemia, including seizures and stroke [3]. The American College of Rheumatology diagnostic criteria for arteritis of Takayasu have a 97.8% specificity and a 90.5% sensitivity when three or more criteria are present [4]. The six criteria are presented in Table 1.

**Table 1:** The American College of Rheumatology diagnostic criteria for Takayasu’s arteritis.

The American College of Rheumatology diagnostic criteria for Takayasu’s arteritis
Age of onset less than or equal to 40 years
Claudication of an extremity
Decreased brachial artery pulse
More than 10 mmHg difference in systolic blood pressure between upper limbs
Bruit over the subclavian arteries or the aorta
Arteriographic evidence of narrowing or occlusion of the entire aorta, its primary branches, or large arteries in the proximal upper or lower extremities

As discussed in this study, our patient met three out of the six criteria and was therefore diagnosed with Takayasu's arteritis. This case shows that an elevated ESR is not always present with active Takayasu's arteritis. Although her CRP and ESR were normal, she was referred to as having active disease due to various systemic features, such as carotid bruit and stenotic vascular lesions. Early recognition of this condition through a comprehensive physical examination and history is crucial.

Although it is generally considered that a normal CRP and ESR are not enough to exclude an individual from having active disease, it is essential to note that using these markers alone can lead to delays in treatment. It is also worth noting that although ESR is the most commonly used clinical monitoring tool, reliance on ESR to assess active Takayasu's arteritis (TAK) can lead to delayed treatment, as it has a 72% sensitivity of only 56% specificity [5].

A study conducted in South Korea revealed that the proportion of cerebral infarction in Takayasu's arteritis was lower in females, and most stroke events were noted in males. Over half of the stroke events occurred within six months after the diagnosis in males compared to after three years in females [6]. Above-stated findings highlight the rarity of stroke as the initial presentation of this disease.

TAK is known as a "pulseless disease" and there is expected to be inequality in blood pressure and pulse in different limbs. Still, Mishra et al. in their study found that patients with Takayasu's arteritis who presented with stroke/transient ischemic attack (TIA) had a lower incidence of blood pressure and pulse inequality compared to those without stroke/TIA. They also found that the patients with stroke or TIA were likelier to be older [7].

The study conducted by Russo and Katsicas revealed that patients with Takayasu's arteritis benefit from immunosuppressant therapy and glucocorticoids [8]. Currently, no robust clinical trials provide a definitive conclusion regarding the treatment of Takayasu's arteritis. In our case, the patient showed improvement after taking medications, such as aspirin, methotrexate, and oral glucocorticoids. Due to the active stage of the disease, any kind of vascular surgical intervention was not possible in our case [9].

## Conclusions

In young stroke patients with no known cardiovascular risk factors, various differential diagnoses, such as those involving inflammatory vasculopathy, should be considered. The significance of this study lies in the fact that Takayasu's arteritis is frequently overlooked in stroke cases, and even in early and active Takayasu's arteritis, the ESR level may be normal; therefore, its normality should not lead to false reassurance.
